# Volatile‐Driven Antioxidant Activity of Pomelo Peel Essential Oil: Bridging Chemical Composition and Cellular Redox Modulation

**DOI:** 10.1155/bri/7715893

**Published:** 2026-07-18

**Authors:** Pratchaya Kaewkaen

**Affiliations:** ^1^ Animal Cognitive Neuroscience Laboratory (ACoN) Unit, Burapha University, Chonburi, Thailand, buu.ac.th; ^2^ Department of Research and Applied Psychology, Faculty of Education, Burapha University, Chonburi, Thailand, buu.ac.th

**Keywords:** cytoprotection, GC–MS, lipid peroxidation, oxidative stress, pomelo peel essential oil, ROS

## Abstract

Pomelo (*Citrus maxima*) peel essential oil is a volatile‐terpenoid‐rich plant extract that may exhibit antioxidant‐associated effects in biological systems. However, the relationship among its chemical composition, conventional antioxidant capacity, and cellular effects under oxidative stress remains insufficiently defined. This study integrated gas chromatography–mass spectrometry (GC–MS) profiling with chemical antioxidant assays and an SH‐SY5Y cell‐based oxidative‐stress model. Under the selected analytical and data‐processing conditions, limonene was the only compound confidently identified and integrated by GC–MS. In chemical assays, pomelo peel essential oil showed moderate DPPH radical‐scavenging and ferric‐reducing activities compared with the hydrophilic reference antioxidants Trolox and ascorbic acid. Cellular effects were evaluated separately in SH‐SY5Y cells exposed to H_2_O_2_‐induced oxidative stress. Pomelo peel essential oil was tested at 25, 50, and 100 μg/mL using cotreatment and pretreatment protocols. In the cotreatment model, cells were exposed simultaneously to the essential oil and H_2_O_2_, whereas in the pretreatment model, cells were incubated with the essential oil before H_2_O_2_ challenge. Pomelo peel essential oil attenuated intracellular ROS accumulation and lipid peroxidation and improved cell viability, with clearer effects at 50 and 100 μg/mL. Pretreatment generally produced stronger cytoprotective effects than cotreatment. These findings indicate that conventional chemical antioxidant assays alone may not fully predict cellular antioxidant‐associated effects, particularly for lipophilic essential oils. Pomelo peel essential oil therefore showed cytoprotective activity in an SH‐SY5Y oxidative‐stress model, although the contributing constituents and underlying molecular mechanisms require further investigation.

## 1. Introduction

Pomelo (*Citrus maxima*) is widely cultivated in tropical and subtropical regions and represents a major source of agroindustrial waste, particularly in the form of peel biomass. Increasing interest has been directed toward the valorization of citrus by‐products due to their high content of bioactive compounds, including essential oils rich in volatile terpenoids [1, 2]. Previous studies of plant‐derived essential oils have similarly combined compositional profiling with antioxidant assays to examine the relationship between chemical composition and biological activity [3]. Among these, limonene is typically the dominant constituent; however, growing evidence indicates that the biological activity of essential oils may arise from complex interactions among multiple components rather than from a single major compound alone [4, 5].

Oxidative stress is a key mechanism involved in cellular injury and neurodegenerative processes. It occurs when reactive oxygen species (ROS), such as superoxide anion (O_2_•^−^), hydrogen peroxide (H_2_O_2_), and hydroxyl radicals (•OH), exceed the capacity of endogenous antioxidant defenses. Excessive ROS can oxidize lipids, proteins, and nucleic acids, ultimately impairing cellular function and viability [6, 7]. Among these downstream events, lipid peroxidation is particularly relevant because neuronal membranes are rich in polyunsaturated fatty acids and are highly vulnerable to oxidative damage [8].

Conventional chemical assays, including DPPH and ferric reducing antioxidant power (FRAP), provide useful estimates of radical‐scavenging or reducing capacity under simplified reaction conditions. However, these assays may not fully predict antioxidant‐associated effects in complex biological or lipid‐rich systems, particularly for hydrophobic compounds such as essential oils and monoterpenes [5, 9]. Because volatile terpenoids may preferentially partition into lipid‐rich environments, complementary cellular assays are needed to evaluate their potential effects under oxidative‐stress conditions [10, 11].

In neuronal systems, oxidative stress is particularly relevant due to high oxygen consumption, abundant polyunsaturated lipids, and limited regenerative capacity. Human neuroblastoma SH‐SY5Y cells provide a well‐established in vitro model for studying neuronal oxidative damage and cytoprotection. Exposure to hydrogen peroxide (H_2_O_2_) induces intracellular ROS generation, lipid peroxidation, and cell death, thereby mimicking key features of oxidative stress‐related neuronal injury [12].

Therefore, the present study aimed to (i) characterize the composition of pomelo peel essential oil using gas chromatography–mass spectrometry (GC–MS), (ii) evaluate its antioxidant activity using conventional chemical assays, including DPPH and FRAP, and (iii) investigate its cellular antioxidant‐associated and cytoprotective effects in SH‐SY5Y cells under H_2_O_2_‐induced oxidative stress. Particular emphasis was placed on the relationship among ROS accumulation, lipid peroxidation, and cell viability. By integrating chemical and cellular approaches, this study seeks to bridge the gap between essential‐oil compositional analysis and biological function, and to clarify the potential role of pomelo peel essential oil in cellular redox modulation.

## 2. Materials and Methods

### 2.1. Plant Material and Essential‐Oil Extraction

Pomelo fruits (*Citrus maxima*), Prachinburi cultivar, were harvested from Si Mahosot District, Prachinburi Province, Thailand, at commercial maturity (approximately 160 days after fruit set). Fruits with a circumference greater than 25 cm were selected. Only fruits that were uniform in size, shape, and maturity and free from visible mechanical damage, disease symptoms, and insect infestation were included. Samples were randomly collected for analysis. Because the citrus peel, particularly the flavedo, is the principal reservoir of volatile terpenes and other essential‐oil constituents, the outer peel was used as the extraction material [1, 13, 14].

After harvest, the fruits were washed thoroughly with clean water to remove adhering impurities and surface contaminants. The flavedo was manually separated from the underlying albedo and cut into small pieces to increase exposed surface area and facilitate volatile release during distillation. The peel was processed immediately after preparation to minimize compositional changes associated with prolonged holding prior to extraction, as citrus peel composition and aromatic properties are known to vary with tissue type and maturity stage [13, 15].

Essential oil was extracted by steam distillation. This method was selected because steam‐based distillation remains one of the most widely applied approaches for essential‐oil isolation and is suitable for recovering volatile constituents from citrus peel while limiting excessive thermal deterioration when compared with harsher extraction conditions [16, 17]. Briefly, the prepared pomelo flavedo was placed in the distillation chamber above a boiling‐water reservoir. Steam generated from the heated water passed through the plant matrix, promoting disruption of oil glands and entrainment of volatile compounds. The vapor mixture was then transferred to a condenser and cooled to obtain a liquid distillate consisting of an aqueous phase and an oil phase [16, 18].

The distillate was collected and allowed to separate by immiscibility and density difference. The essential‐oil layer was isolated using a separatory funnel, while the aqueous distillate (hydrosol) was collected separately. This phase‐separation behavior is characteristic of distilled essential oils and hydrosols obtained from aromatic plant materials [1, 16].

The recovered essential oil was transferred to amber glass vials, tightly sealed, and stored under refrigerated conditions (4°C‐5°C) until chemical and biological analyses. Protection from light and low‐temperature storage were applied to reduce postextraction compositional instability of volatile constituents during storage [19]. Residual moisture was removed using anhydrous sodium sulfate before analysis.

### 2.2. GC–MS Analysis of Essential Oil

The volatile composition of pomelo (*Citrus maxima*) peel essential oil was analyzed using GC–MS. The analysis was performed on an Agilent 6890N gas chromatograph coupled with a 5973 inert mass selective detector (Agilent Technologies, USA). Separation was achieved using an HP‐5 capillary column (30 m × 0.25 mm i.d., film thickness 0.25 μm). Helium was used as the carrier gas at a constant flow rate of 1.0 mL/min. The injection was performed in split mode (50:1), with an injector temperature of 250°C. The oven temperature was programmed as follows: initial temperature at 50°C (held for 2 min), increased to 200°C at a rate of 10°C/min (held for 3 min), and then increased to 260°C at a rate of 15°C/min (held for 20 min). The detector temperature was set at 270°C. Mass spectra were acquired in electron ionization (EI) mode with a scan range of 30–500 amu. The solvent delay was set at 3 min. Ethanol (HPLC grade) was used as the solvent for sample preparation. For GC–MS analysis, pomelo peel essential oil was diluted in HPLC‐grade ethanol to a final concentration of 10 mg/mL. The diluted sample was vortexed thoroughly and filtered through a 0.22‐μm PTFE syringe filter before injection. An aliquot of 1 μL was injected into the GC–MS system using a split ratio of 50:1. These dilution and injection conditions were selected to obtain a stable chromatographic signal for the major volatile component while minimizing the risk of detector saturation. Compound identification was performed by comparing the obtained mass spectra with those in the Wiley 7n.1 mass spectral library. Only compounds with high matching quality were considered for identification.

For compositional interpretation, peak area normalization was applied only to peaks that passed the predefined identification and integration criteria. Therefore, a relative area of 100.00% for dl‐limonene denotes 100.00% of the confidently integrated peak area in the processed chromatographic report, not the complete chemical composition of the essential oil. Minor low‐intensity signals that did not meet the criteria for reliable assignment were not quantified or used to infer biological activity.

### 2.3. Determination of DPPH Radical‐Scavenging Activity

The DPPH radical‐scavenging activity of pomelo (Citrus maxima) peel essential oil was determined according to a previously reported method with minor modifications. Pomelo peel essential oil was initially dispersed in methanol containing 0.5% (v/v) Tween 80 and serially diluted with methanol to the required concentrations. The sample solutions were prepared at concentrations ranging from 0.50 to 8.00 mg/mL before mixing with the DPPH solution, corresponding to final concentrations of 0.25 to 4.00 mg/mL in the reaction mixture. Trolox, ascorbic acid, and limonene were prepared in methanol as reference compounds. A sample blank containing the same concentration of essential‐oil solution without DPPH was used to correct for background absorbance caused by sample turbidity or intrinsic color. Briefly, 0.5 mL of each sample solution was mixed with 0.5 mL of 0.4 mM DPPH solution in methanol. The reaction mixture was vortexed thoroughly and incubated in the dark at room temperature for 30 min.

After incubation, the absorbance was measured at 517 nm using a UV–Vis spectrophotometer. A control solution containing DPPH and methanol without sample was prepared, while a sample blank (sample solution without DPPH) was used to correct for background absorbance.

The radical‐scavenging activity was calculated as follows:

(1) % inhibition=A_control−A_sample−A_blankA_control×100,

where A_control is the absorbance of the DPPH solution without sample, A_sample is the absorbance of the reaction mixture containing the sample and DPPH, and A_blank is the absorbance of the sample solution without DPPH.

The antioxidant activity was expressed as percentage inhibition and IC50 value, where the IC50 was defined as the concentration of sample required to inhibit 50% of the DPPH radicals. All measurements were performed in triplicate, and results were expressed as mean ± standard deviation (SD). Trolox and ascorbic acid were used as reference antioxidants for comparison.

### 2.4. Determination of FRAP

The FRAP assay was performed according to a previously reported method with minor modifications. The FRAP reagent was freshly prepared by mixing 25 mL of 300 mM acetate buffer (pH 3.6), 2.5 mL of 10 mM 2,4,6‐tripyridyl‐s‐triazine (TPTZ) solution in 40 mM HCl, and 2.5 mL of 20 mM FeCl_3_·6H_2_O solution. For the FRAP assay, pomelo peel essential oil was dissolved in methanol to prepare a stock solution at a concentration of 10 mg/mL and vortexed thoroughly before use. Trolox was prepared in methanol, whereas ascorbic acid was prepared in distilled water. The final methanol concentration was maintained consistently across all sample, standard, and blank reactions. A reagent blank and a sample blank were included to correct for baseline absorbance and sample‐related background signals. Briefly, 10 μL of the essential‐oil stock solution was diluted in 1 mL of methanol and mixed with 1.8 mL of freshly prepared FRAP reagent. The reaction mixture was incubated at 37°C for 10 min, and absorbance was measured at 593 nm. A reagent blank containing FRAP solution without sample was used for baseline correction. The antioxidant capacity was calculated from a standard calibration curve and expressed as μmol Fe^2+^ equivalents per gram of sample (μmol Fe^2+^/g) and/or μmol Trolox equivalent per gram of sample (μmol TE/g). All measurements were performed in triplicate, and the results were expressed as mean ± SD.

### 2.5. Cell‐Line Validation

The authenticated human neuroblastoma SH‐SY5Y cell line (ATCC CRL‐2266; RRID: CVCL_0019) was used in this study. Cell‐line identity was confirmed by short tandem repeat (STR) profiling performed within the past 3 years, with the resulting profile compared against reference databases. Mycoplasma contamination was routinely assessed using PCR‐based testing, and all cultures used in the experiments were confirmed to be mycoplasma‐free.

### 2.6. Determination of Cytoprotective Activity

Pomelo peel essential oil was dissolved in ethanol and diluted with culture medium to final concentrations of 25, 50, and 100 μg/mL. The final ethanol concentration in all treatment groups, including the vehicle control, was maintained below 0.5% (v/v). SH‐SY5Y cells were seeded at a density of 3 × 10^4^ cells/cm^2^ and incubated for 24 h before treatment. Oxidative stress was induced using H_2_O_2_ at a final concentration of 500 μM, which reduced cell viability to approximately 50%. Two treatment protocols were evaluated. In the cotreatment model, cells were simultaneously exposed to pomelo peel essential oil and H_2_O_2_ for 24 h. In the pretreatment model, cells were preincubated with pomelo peel essential oil for 6 h, followed by H_2_O_2_ exposure for an additional 24 h. Vehicle‐control cells received culture medium containing the corresponding final ethanol concentration. Essential‐oil‐only control groups were included to confirm that the tested concentrations of pomelo peel essential oil did not induce cytotoxicity in the absence of H_2_O_2_.

For each independent experiment, solvent‐matched vehicle controls were run in parallel to account for the final ethanol concentration. Essential‐oil‐only controls at 25, 50, and 100 μg/mL were also included under the corresponding exposure conditions to verify that the tested concentrations did not produce cytotoxicity in the absence of H_2_O_2_. These control groups were used as assay‐validity controls for interpreting cytoprotective responses under oxidative‐stress conditions.

Cell viability was assessed using the MTT assay. Following treatment, cells were incubated with MTT solution at a final concentration of 0.5 mg/mL for 4 h at 37°C. The resulting formazan crystals were dissolved in dimethyl sulfoxide (DMSO), and absorbance was measured at 570 nm using a microplate reader. Cell viability was expressed as a percentage of the untreated control group. All experiments were performed independently three times, and the results are expressed as mean ± SD.

### 2.7. Determination of Intracellular ROS

Intracellular ROS levels were determined using the carboxy‐H_2_DCFDA fluorescent probe. SH‐SY5Y cells were treated with pomelo peel essential oil at 100 μg/mL under the cotreatment and pretreatment conditions described above. In the cotreatment model, cells were exposed simultaneously to pomelo peel essential oil and H_2_O_2_ (500 μM) for 24 h. In the pretreatment model, cells were preincubated with pomelo peel essential oil for 6 h before exposure to H_2_O_2_ (500 μM) for 24 h.

Following treatment, cells were washed gently with phosphate‐buffered saline (PBS) and incubated with carboxy‐H_2_DCFDA (10 μM in serum‐free medium) for 30 min at 37°C in the dark. Excess probe was removed by washing with PBS, and fluorescence intensity was measured at excitation and emission wavelengths of 485 and 528 nm, respectively. Intracellular ROS levels were expressed relative to the untreated control group. All experiments were performed in triplicate and are reported as mean ± SD from three independent experiments.

Fluorescence readings were corrected for background signal and expressed relative to the untreated control group. Direct normalization to protein content, DNA content, or viable cell number was not performed; therefore, ROS fluorescence was interpreted as a relative index of intracellular oxidative status rather than an absolute rate of ROS production per cell.

### 2.8. Determination of Lipid Peroxidation

Intracellular lipid peroxidation was quantified using the lipophilic fluorescent probe C11‐BODIPY 581/591, which exhibits an oxidation‐dependent fluorescence shift and is widely used for detecting membrane lipid peroxidation in neuronal and ferroptosis‐related models [20]. SH‐SY5Y cells were seeded into black 96‐well plates and allowed to attach for 24 h under standard culture conditions. Pomelo (*Citrus maxima*) peel essential oil was dissolved in ethanol and diluted with culture medium to the desired concentrations, with the final ethanol concentration maintained below 0.5% (v/v) in all groups, including the vehicle control. The experimental design followed the same cotreatment and pretreatment conditions used in the intracellular ROS assay. Following treatment under oxidative stress conditions induced by H_2_O_2_, the cells were washed with PBS and incubated with C11‐BODIPY 581/591 (5 μM in serum‐free medium) for 30 min at 37°C in the dark. The probe incorporates into cellular membranes and is oxidized by lipid peroxyl radicals, resulting in a fluorescence shift from red to green [21, 22].

After incubation, excess dye was removed by washing with PBS, and fluorescence intensity was measured using a microplate reader. The oxidized (green) and reduced (red) forms were detected at excitation/emission wavelengths of 485/520 nm and 560/590 nm, respectively. Lipid peroxidation was expressed as the ratio of green to red fluorescence, reflecting the extent of probe oxidation [22]. To minimize potential interference from essential‐oil autofluorescence, background fluorescence from treated cells without probe was subtracted. All experiments were performed in triplicate, and the results were expressed as mean ± SD.

The C11‐BODIPY oxidation index was calculated as the background‐corrected green fluorescence intensity divided by the background‐corrected red fluorescence intensity [F_green (Ex 485 nm/Em 520 nm)/F_red (Ex 560 nm/Em 590 nm)]. Matched no‐probe wells for each treatment condition, including essential‐oil‐treated wells, were used to correct for background fluorescence and potential essential‐oil‐associated autofluorescence before ratio calculation.

### 2.9. Statistical Analysis

All experiments were performed using at least three independent biological replicates, and the results are expressed as mean ± SD. Statistical analyses were conducted using SPSS Version 27 (IBM Corp., Armonk, NY, USA). Differences among multiple groups were analyzed using one‐way analysis of variance (ANOVA), followed by Tukey’s post hoc test to determine pairwise comparisons between group means. A value of *p* < 0.05 was considered statistically significant. Prior to analysis, data distribution and homogeneity of variance were evaluated using the Shapiro–Wilk test and Levene’s test, respectively, where applicable. When necessary, data were transformed to meet the assumptions of parametric analysis.

## 3. Results

### 3.1. Volatile Composition of Pomelo Peel Essential Oil

**FIGURE 1:**
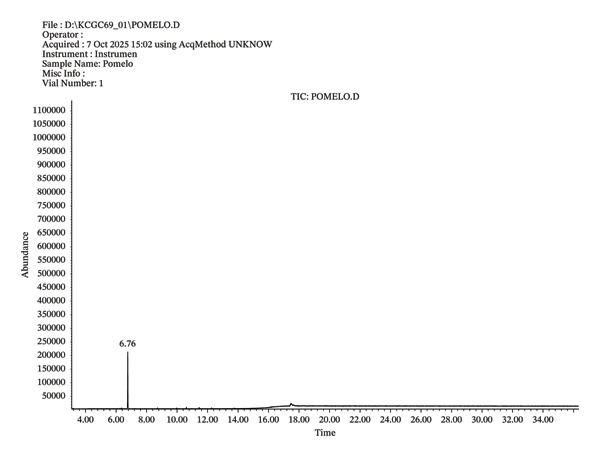
Total ion chromatogram (TIC) of pomelo (*Citrus maxima*) peel essential oil obtained by GC–MS analysis. A dominant peak corresponding to dl‐limonene was observed at a retention time of 6.76 min.

**FIGURE 2:**
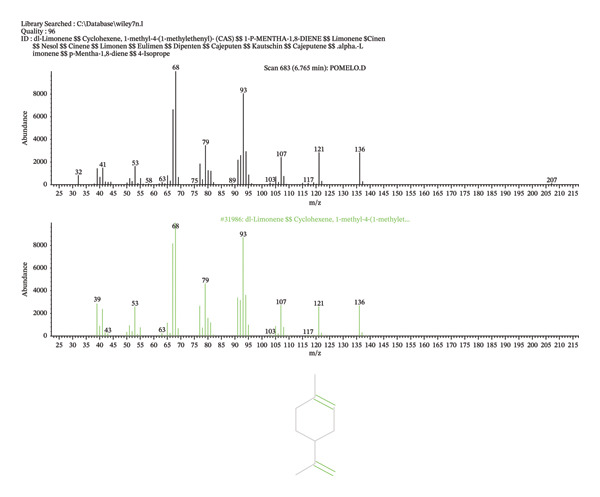
GC–MS mass spectrum and library matching of limonene identified in pomelo (*Citrus maxima*) peel essential oil. Compounds were identified based on comparison of mass spectra with library databases. Relative composition (%) was calculated from the peak area normalization of the total ion chromatogram. Under the selected GC–MS analytical and data‐processing conditions, limonene was the only compound confidently identified and integrated. The value of 100.00% refers to the relative area of the integrated peak in the processed chromatographic report and should not be interpreted as the complete chemical composition of the essential oil.

GC–MS analysis of pomelo peel essential oil revealed a dominant peak at a retention time of 6.765 min. This peak was identified as dl‐limonene by comparison with the Wiley 7n.1 mass spectral library, with a match quality of 96%. Under the selected GC–MS conditions and data‐processing threshold, dl‐limonene was the only compound confidently identified and integrated in the processed chromatographic report. Therefore, the value of 100.00% refers only to the relative area of the integrated peak under the applied processing conditions and should not be interpreted as evidence that the essential oil consisted exclusively of limonene. Although low‐intensity signals were observed at higher retention times, these peaks were not sufficiently resolved or confidently identified. Therefore, they were not assigned to specific compounds or used for compositional interpretation in the present study (Figures 1 and 2).

### 3.2. DPPH Radical‐Scavenging Activity of Pomelo (*Citrus maxima*) Peel Essential Oil

The antioxidant activity of pomelo (*Citrus maxima*) peel essential oil was evaluated using the DPPH radical‐scavenging assay and compared with standard antioxidants, including Trolox, ascorbic acid, and pure limonene. The results are expressed as IC_50_ values and Trolox equivalent antioxidant capacity (TEAC).

The essential oil exhibited moderate radical‐scavenging activity, with an IC_50_ value of 1.85 ± 0.07 mg/mL and a TEAC value of 112.4 ± 3.6 μmol TE/g sample. In comparison, Trolox and ascorbic acid showed significantly stronger activity, with IC_50_ values of 0.021 ± 0.002 mg/mL and 0.018 ± 0.001 mg/mL, respectively. Pure limonene demonstrated relatively low activity (IC_50_ = 3.92 ± 0.15 mg/mL), indicating limited radical‐scavenging capacity.

Although limonene was the only compound confidently integrated under the selected GC–MS conditions, the antioxidant behavior of the whole essential oil may not be explained solely by limonene. Because minor constituents were not confidently identified or quantified, any contribution of trace constituents or component interactions remains hypothetical. The DPPH radical‐scavenging results are summarized in Table 1.

**TABLE 1:** DPPH radical scavenging activity of pomelo (*Citrus maxima*) peel essential oil and reference compounds.

Sample	IC_50_ (mg/mL)	TEAC (μmol TE/g)
Pomelo peel essential oil	1.85 ± 0.07	112.4 ± 3.6
Trolox	0.021 ± 0.002	n.a.
Ascorbic acid	0.018 ± 0.001	n.a.
Limonene	3.92 ± 0.15	35.7 ± 2.1

*Note:* Values are expressed as mean ± SD (*n* = 3 independent determinations). IC_50_ is expressed as mg/mL and represents the concentration required to inhibit 50% of DPPH radicals. TEAC is expressed as μmol Trolox equivalent per gram of sample (μmol TE/g). Trolox, ascorbic acid, and limonene were used as reference compounds.

Abbreviation: n.a., not applicable.

### 3.3. FRAP of Pomelo (*Citrus maxima*) Peel Essential Oil

The FRAP of pomelo (*Citrus maxima*) peel essential oil was evaluated and compared with standard antioxidants. The essential oil exhibited moderate reducing capacity, with a value of 98.6 ± 4.2 μmol Fe^2+^/g sample, corresponding to 105.3 ± 3.8 μmol TE/g sample. In contrast, ascorbic acid and Trolox showed significantly higher reducing power, with TE values of 1125.4 ± 30.2 and 1000.0 ± 25.0 μmol TE/g, respectively. Pure limonene demonstrated relatively low reducing activity (28.4 ± 1.6 μmol Fe^2+^/g), indicating limited electron‐donating capacity as shown in Table 2.

**TABLE 2:** Ferric reducing antioxidant power (FRAP) of pomelo (*Citrus maxima*) peel essential oil and reference compounds.

Sample	FRAP (μmol Fe^2+^/g sample)	FRAP (μmol TE/g sample)
Pomelo peel essential oil	98.6 ± 4.2	105.3 ± 3.8
Trolox	n.a.	1000.0 ± 25.0
Ascorbic acid	n.a.	1125.4 ± 30.2
Limonene	28.4 ± 1.6	32.7 ± 2.0

*Note:* Values are expressed as mean ± SD (*n* = 3 independent determinations). FRAP values are expressed as μmol Fe^2+^ equivalents per gram of sample (μmol Fe^2+^/g sample) and/or μmol Trolox equivalents per gram of sample (μmol TE/g sample). Trolox, ascorbic acid, and limonene were used as reference compounds.

Abbreviation: n.a., not applicable.

### 3.4. Cytoprotective Effect of Pomelo (*Citrus maxima*) Peel Essential Oil Against H_2_O_2_‐Induced Cytotoxicity in SH‐SY5Y Cells

Treatment with pomelo peel essential oil improved cell viability under oxidative‐stress conditions. In the cotreatment model, cell viability was significantly higher than that in the H_2_O_2_‐treated group (*p* < 0.05), although it remained lower than that in the untreated control group. Pretreatment with pomelo peel essential oil resulted in higher cell viability than the H_2_O_2_‐treated and cotreatment groups (*p* < 0.05). These findings indicate a greater cytoprotective effect under the pretreatment condition (Table 3).

**TABLE 3:** Cytoprotective effect of pomelo (*Citrus maxima*) peel essential oil against H_2_O_2_‐induced cytotoxicity in SH‐SY5Y cells.

Treatment group	Cell viability (%)	Significance
Control	100.0 ± 0.0	a
H_2_O_2_ (500 μM)	50.0 ± 3.0	c
Pomelo peel essential oil [100 μg/mL] + H_2_O_2_, cotreatment 24 h	65.0 ± 2.5	b
Pomelo peel essential oil [100 μg/mL] pretreatment 6 h + H_2_O_2_ 24 h	80.0 ± 2.8	a

*Note:* Data are expressed as mean ± SD from three independent experiments (*n* = 3). Cell viability was measured using the MTT assay and expressed as a percentage of the untreated control group. In the cotreatment model, SH‐SY5Y cells were exposed simultaneously to pomelo peel essential oil (100 μg/mL) and H_2_O_2_ (500 μM) for 24 h. In the pretreatment model, cells were preincubated with pomelo peel essential oil (100 μg/mL) for 6 h before exposure to H_2_O_2_ (500 μM) for 24 h. Different superscript letters indicate statistically significant differences among groups (*p* < 0.05). Vehicle and essential‐oil‐only control groups were included in parallel as assay‐validity controls and showed no cytotoxicity under the tested conditions.

Solvent‐matched vehicle controls did not show a detectable reduction in cell viability relative to untreated controls under the present assay conditions. Essential‐oil‐only exposure at 25, 50, and 100 μg/mL also did not show cytotoxicity in the absence of H_2_O_2_; thus, the observed changes in viability were interpreted as responses to oxidative challenge rather than solvent‐ or essential‐oil‐induced toxicity alone.

### 3.5. Intracellular ROS Levels

**FIGURE 3:**
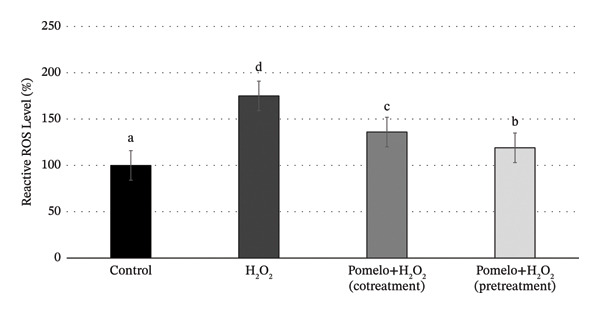
Effect of pomelo peel essential oil on intracellular ROS levels in SH‐SY5Y cells exposed to H_2_O_2_‐induced oxidative stress. SH‐SY5Y cells were treated with H_2_O_2_ (500 μM) for 24 h. In the cotreatment model, cells were simultaneously treated with pomelo peel essential oil (100 μg/mL) and H_2_O_2_ for 24 h. In the pretreatment model, cells were preincubated with pomelo peel essential oil for 6 h before H_2_O_2_ exposure for 24 h. Intracellular ROS levels were measured using carboxy‐H_2_DCFDA and expressed as percentage of the untreated control group. Data are expressed as mean ± SD (*n* = 3 independent experiments). Different letters indicate statistically significant differences among groups (*p* < 0.05).

Intracellular ROS production in SH‐SY5Y cells was assessed using the carboxy‐H_2_DCFDA assay under oxidative‐stress conditions. Exposure to H_2_O_2_ significantly increased ROS levels compared with the untreated control group (*p* < 0.01), reaching approximately 175% of the control value and confirming the induction of oxidative stress. Cotreatment with pomelo peel essential oil attenuated intracellular ROS accumulation to approximately 135% of the control value (*p* < 0.05 versus the H_2_O_2_‐treated group). Pretreatment produced a greater reduction, decreasing ROS levels to approximately 120% of the control value (*p* < 0.05 versus the H_2_O_2_‐treated group). However, ROS levels in both essential‐oil treatment groups remained higher than those in the untreated control group. These findings indicate that pomelo peel essential oil attenuated H_2_O_2_‐induced intracellular ROS accumulation, with a greater effect observed under the pretreatment condition (Figure 3 and Table 4).

**TABLE 4:** Effect of pomelo (*Citrus maxima*) peel essential oil on intracellular ROS levels in H_2_O_2_‐treated SH‐SY5Y cells.

Treatment group	ROS level (% of control)	Significance
Control	100.0 ± 4.8	a
H_2_O_2_ (500 μM)	176.3 ± 9.7	d
Pomelo peel essential oil [100 μg/mL] + H_2_O_2_, cotreatment 24 h	136.8 ± 7.2	c
Pomelo peel essential oil [100 μg/mL] pretreatment 6 h + H_2_O_2_ 24 h	121.4 ± 6.1	b

*Note:* Data are expressed as mean ± SD (*n* = 3 independent experiments). ROS levels were measured using carboxy‐H_2_DCFDA and expressed as percentage of the untreated control group. In the cotreatment model, cells were simultaneously exposed to pomelo peel essential oil and H_2_O_2_ for 24 h. In the pretreatment model, cells were preincubated with pomelo peel essential oil for 6 h before H_2_O_2_ exposure for 24 h. Different letters indicate statistically significant differences among groups (*p* < 0.05). ROS fluorescence values were background‐corrected and expressed relative to untreated controls; no direct normalization to protein, DNA, or viable cell number was performed.

Because the carboxy‐H_2_DCFDA signal was expressed relative to the untreated control and was not normalized to viable cell number, protein, or DNA content, these data are presented as relative fluorescence‐based changes in intracellular oxidative status. The ROS results were therefore interpreted together with cell‐viability data rather than as absolute ROS production per cell.

### 3.6. Effects of Pomelo Peel Essential Oil on Lipid Peroxidation

**FIGURE 4:**
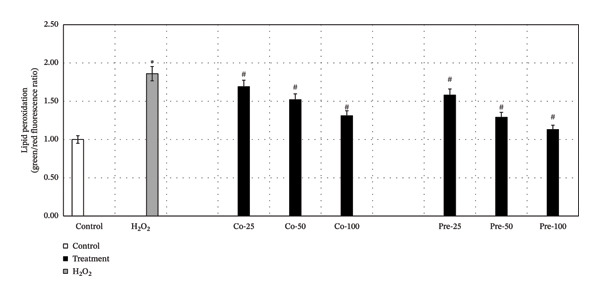
Effect of pomelo peel essential oil on lipid peroxidation in SH‐SY5Y cells exposed to H_2_O_2_‐induced oxidative stress. Lipid peroxidation was assessed using C11‐BODIPY 581/591 and expressed as the oxidized green/reduced red fluorescence ratio. Co‐25, Co‐50, and Co‐100 indicate cotreatment with pomelo peel essential oil at 25, 50, and 100 μg/mL, respectively, together with H_2_O_2_ (500 μM) for 24 h. Pre‐25, Pre‐50, and Pre‐100 indicate pretreatment with pomelo peel essential oil at 25, 50, and 100 μg/mL, respectively, for 6 h before H_2_O_2_ exposure for 24 h. Data are expressed as mean ± SD from three independent experiments (*n* = 3). ^∗^ *p* < 0.05 versus the untreated control group; ^#^ *p* < 0.05 versus the H_2_O_2_‐treated group. The green/red ratio was calculated after subtraction of matched no‐probe background fluorescence for each treatment condition, including essential‐oil‐treated wells, to minimize interference from treatment‐related autofluorescence.

Lipid peroxidation in SH‐SY5Y cells was quantified using the C11‐BODIPY 581/591 fluorescent probe under H_2_O_2_‐induced oxidative‐stress conditions. As shown in Figure 4, exposure to H_2_O_2_ significantly increased the green/red fluorescence ratio compared with the untreated control group (1.86 ± 0.12 vs. 1.00 ± 0.08, *p* < 0.05), indicating enhanced lipid peroxidation. In the cotreatment model, pomelo peel essential oil reduced lipid peroxidation in a concentration‐dependent manner. Significant reductions were observed at 25, 50, and 100 μg/mL, with fluorescence ratios of 1.69 ± 0.11, 1.52 ± 0.10, and 1.31 ± 0.09, respectively, compared with the H_2_O_2_‐treated group (*p* < 0.05).

In the pretreatment model, pretreatment with the essential oil for 6 h prior to H_2_O_2_ exposure significantly attenuated lipid peroxidation at all tested concentrations. The fluorescence ratios were 1.58 ± 0.10, 1.29 ± 0.08, and 1.12 ± 0.07 at 25, 50, and 100 μg/mL, respectively (*p* < 0.05 vs. H_2_O_2_). At corresponding concentrations, the pretreatment condition yielded numerically lower fluorescence ratios than the cotreatment condition. Overall, these findings indicate that pomelo peel essential oil attenuated H_2_O_2_‐induced lipid peroxidation in SH‐SY5Y cells in a concentration‐dependent manner (Figure 4).

## 4. Discussion

GC–MS analysis identified limonene as the only compound confidently integrated under the selected analytical and data‐processing conditions. This finding is consistent with previous reports, indicating that limonene is commonly a major monoterpene in citrus peel essential oils [1, 14]. However, the reported 100.00% integrated peak area should not be interpreted as evidence that the essential oil consisted exclusively of limonene. Low‐intensity signals were observed but could not be reliably resolved, identified, or quantified. Accordingly, the possible contribution of trace constituents or interactions among multiple components remains hypothetical. Future studies should compare the whole essential oil with pure limonene at equivalent concentrations and, where feasible, evaluate reconstructed mixtures of identified constituents.

A recent study using SH‐SY5Y cells also reported that a phytochemical‐rich preparation attenuated hydrogen peroxide‐associated oxidative injury [23]. Nevertheless, the magnitude and biological basis of such protective responses may vary according to chemical composition, exposure conditions, and the endpoints examined. Accordingly, the present findings should be interpreted specifically in relation to pomelo peel essential oil and the redox‐related outcomes assessed, rather than as evidence of a generalized neuroprotective mechanism. Pomelo peel essential oil demonstrated moderate antioxidant capacity in the DPPH and FRAP assays compared with the hydrophilic reference antioxidants. These assays are conducted under simplified chemical conditions and primarily reflect radical‐scavenging or reducing capacity [5, 9]. Lipophilic compounds, including monoterpenes, may show limited apparent activity because of restricted solubility and reduced interaction with radical species in homogeneous reaction systems. Accordingly, DPPH and FRAP results may not fully predict antioxidant‐associated effects in biological or lipid‐rich environments [10].

The present chemical antioxidant profile may also be interpreted in relation to essential‐oil chemotypes reported in other aromatic species. Previous studies on *Minthostachys mollis* essential oil have shown that antioxidant performance in lipid oxidation systems can vary according to chemical composition, chemotype, oxidative substrate, and interaction with other antioxidants. For example, M. mollis essential oil and its combination with tert‐butylhydroquinone (TBHQ) have been evaluated in sunflower oil oxidation models, suggesting that essential oils may influence lipid oxidation even when their direct radical‐scavenging capacity is limited [24]. Similarly, combinations of M. mollis and *Origanum vulgare* essential oils have been investigated in relation to “chain‐breaking” and “termination‐enhancing” antioxidant mechanisms, supporting the view that essential‐oil antioxidant behavior depends strongly on chemical composition and the oxidation model used [25]. Compared with these more chemically complex essential oils, the present pomelo peel essential oil was dominated by dl‐limonene under the selected GC–MS conditions; therefore, direct comparison should be made cautiously.

The moderate DPPH radical‐scavenging activity observed for pomelo peel essential oil may also be compared with studies on other monoterpene‐rich essential oils, particularly oils from the genus Cymbopogon. Citronella, lemongrass, and palmarosa essential oils contain different profiles of monoterpenes and oxygenated terpenoids, and their antioxidant performance can vary depending on both chemical composition and the oxidation model used. A recent study evaluating Cymbopogon essential oils in roasted peanuts reported their potential to delay oxidative processes in food systems, highlighting that antioxidant effectiveness may differ between chemical radical‐scavenging assays and lipid‐rich food oxidation models [26]. In this context, the moderate DPPH activity of pomelo peel essential oil may be related to its limonene‐dominant profile, as hydrocarbon monoterpenes generally show weaker direct radical‐scavenging activity than phenolic antioxidants or some oxygenated terpenoids. Therefore, comparison with citronella and related essential oils supports the interpretation that DPPH results should be considered together with chemical composition and complementary lipid or cellular oxidation assays.

In contrast to the chemical assays, pomelo peel essential oil was associated with clearer cellular antioxidant‐related responses in SH‐SY5Y cells exposed to H_2_O_2_‐induced oxidative stress. Treatment with the essential oil was accompanied by lower intracellular ROS fluorescence, lower C11‐BODIPY oxidation ratios, and higher cell viability relative to H_2_O_2_ exposure alone. These findings should be interpreted as convergent changes across oxidative‐stress‐related endpoints under the present experimental conditions, rather than as evidence that a defined antioxidant pathway was activated. The present data therefore support an association among reduced fluorescence‐based oxidative‐stress indices and improved cell viability, but they do not establish a specific molecular pathway.

Because the cellular experiments were limited to an SH‐SY5Y H_2_O_2_ model and fluorescence‐based oxidative‐stress endpoints, the findings should not be interpreted as definitive evidence of neuroprotection or as proof of a specific membrane‐associated antioxidant mechanism. Rather, they provide evidence of cytoprotection associated with reduced oxidative‐stress‐related readouts in this in vitro model.

Pretreatment with pomelo peel essential oil generally showed a more favorable cytoprotective pattern than cotreatment. This observation may indicate that prior exposure to the essential oil modifies cellular responses to subsequent oxidative challenge; however, this interpretation remains inferential because adaptive signaling pathways were not directly examined. The pattern is compatible with, but does not demonstrate, a preconditioning‐like response. Redox‐sensitive pathways such as the Nrf2/ARE axis may be relevant to future investigation because this pathway regulates antioxidant and detoxifying enzymes, including SOD, CAT, GPx, and HO‐1 [27, 28]. Further experiments, including Nrf2 and HO‐1 protein analysis, antioxidant enzyme assays, and GSH/GSSG measurement, are required before any pathway‐specific conclusion can be drawn.

Because lipid peroxidation is a central feature of ferroptosis, the observed reduction in C11‐BODIPY oxidation may be consistent with reduced ferroptosis‐related lipid damage. However, ferroptosis was not directly demonstrated in this study because key markers such as GPX4 activity, intracellular iron level, lipid hydroperoxide accumulation, and ferroptosis inhibitor rescue were not assessed. Therefore, the relationship between pomelo peel essential oil and ferroptosis‐related pathways should be regarded as a hypothesis requiring further investigation.

Taken together, these findings support an association among reduced intracellular oxidative stress, attenuated membrane lipid peroxidation, and improved cell viability; however, they do not establish a specific molecular pathway.

An important implication of this study is that chemical antioxidant assays alone may not fully predict cellular antioxidant‐associated responses. This interpretation highlights the need to consider physicochemical properties, including lipophilicity, solubility, membrane partitioning, and assay environment, when evaluating essential oils. The concept of compartmentalized antioxidant activity provides a useful framework for interpreting why chemical assay performance and cellular oxidative‐stress readouts may not fully align; however, the present study did not directly measure membrane partitioning or compartment‐specific antioxidant activity [10].

Several limitations should be acknowledged. First, only dl‐limonene was confidently identified and integrated under the selected GC–MS analytical and data‐processing conditions. Although low‐intensity signals were observed, they were not sufficiently resolved or identified to allow reliable quantification. Future studies should optimize GC–MS conditions, including sample concentration, split ratio, integration threshold, and the use of authentic standards, to better identify and quantify trace constituents. Second, the present study was conducted using a single neuronal cell model, SH‐SY5Y cells, under acute H_2_O_2_‐induced oxidative‐stress conditions. Although SH‐SY5Y cells are widely used to investigate neuronal oxidative injury and cytoprotective responses, findings derived from one cell line may not fully capture the biological complexity and heterogeneity of neuronal responses in vivo. Further studies should validate these findings in additional neuronal cell models, primary neuronal cultures, and relevant animal models. Third, mechanistic pathways, including antioxidant enzyme regulation, Nrf2‐related signaling, glutathione metabolism, and ferroptosis‐related processes, were not directly examined and require further investigation. Finally, DCFH‐DA fluorescence may be affected by cell number, cell viability, dye loading efficiency, and intracellular esterase activity. In the present study, ROS fluorescence was expressed relative to the untreated control and interpreted alongside cell‐viability data; however, direct normalization to protein content, DNA content, or viable cell number was not performed. Therefore, the ROS results should be interpreted as relative changes in intracellular oxidative status rather than absolute ROS production per cell.

In addition, vehicle and essential‐oil‐only controls were included to support interpretation of the oxidative‐stress experiments, but the present study was not designed to define the independent pharmacological effects of pomelo peel essential oil under basal conditions. The ROS assay also provides relative fluorescence‐based information and may be influenced by cell number, viability, dye loading, and esterase activity; this limitation should be considered when comparing ROS values across treatment groups.

## 5. Conclusion

In conclusion, pomelo peel essential oil was associated with higher cell viability and lower fluorescence‐based oxidative‐stress readouts in an SH‐SY5Y model of H_2_O_2_‐induced oxidative stress, particularly at 50 and 100 μg/mL. These responses were accompanied by lower relative intracellular ROS fluorescence and reduced C11‐BODIPY green/red fluorescence ratios, with the pretreatment protocol showing a more favorable pattern than cotreatment. The response at 25 μg/mL was weaker and endpoint‐dependent, particularly in the cotreatment condition. The present findings should be interpreted as in vitro evidence of antioxidant‐associated cytoprotection rather than definitive evidence of neuroprotection, membrane‐specific antioxidant action, ferroptosis inhibition, or activation of a confirmed molecular pathway. Further studies are required to identify trace constituents, compare the whole essential oil with pure limonene, and investigate antioxidant enzyme regulation, Nrf2‐related signaling, glutathione status, GPX4 activity, intracellular iron levels, and ferroptosis‐related markers.

## Funding

The author gratefully acknowledges the financial support provided by the Faculty of Education, Burapha University, Thailand, under Research Grant Contract No. 005/2568.

## Conflicts of Interest

The author declares no conflicts of interest.

## Data Availability

The data that support the findings of this study are available from the corresponding author upon reasonable request.

## References

[1] González-Mas M. C. , Rambla J. L. , López-Gresa M. P. , Blázquez M. A. , and Granell A. , Volatile Compounds in *Citrus* Essential Oils: A Comprehensive Review, Frontiers in Plant Science. (2019) 10, 10.3389/fpls.2019.00012. PMC6370709 30804951

[2] Andrade M. A. , Barbosa C. H. , Shah M. A. et al., *Citrus* By-Products: Valuable Source of Bioactive Compounds for Food Applications, Antioxidants (Basel, Switzerland). (2022) 12, no. 1, 10.3390/antiox12010038. PMC9855225 36670900

[3] Yitayeh M. M. and Wassihun A. M. , Chemical Composition and Antibacterial and Antioxidant Activities of Stem Bark Essential Oil and Extracts of *Solanecio gigas* , Biochemistry Research International. (2022) 2022, 10.1155/2022/4900917. PMC9288319 35855890

[4] Bakkali F. , Averbeck S. , Averbeck D. , and Idaomar M. , Biological Effects of Essential Oils--A Review, Food and Chemical Toxicology: An International Journal Published for the British Industrial Biological Research Association. (2008) 46, no. 2, 446–475, 10.1016/j.fct.2007.09.106. 17996351

[5] Amorati R. and Valgimigli L. , Advantages and Limitations of Common Testing Methods for Antioxidants, Free Radical Research. (2015) 49, no. 5, 633–649, 10.3109/10715762.2014.996146. 25511471

[6] Uttara B. , Singh A. V. , Zamboni P. , and Mahajan R. T. , Oxidative Stress and Neurodegenerative Diseases: A Review of Upstream and Downstream Antioxidant Therapeutic Options, Current Neuropharmacology. (2009) 7, no. 1, 65–74, 10.2174/157015909787602823. 19721819 PMC2724665

[7] Ji L. L. and Yeo D. , Oxidative Stress: An Evolving Definition, Faculty reviews. (2021) 10, 10.12703/r/10-13. PMC7894272 33659931

[8] Ayala A. , Muñoz M. F. , and Argüelles S. , Lipid Peroxidation: Production, Metabolism, and Signaling Mechanisms of Malondialdehyde and 4-Hydroxy-2-Nonenal, Oxidative Medicine and Cellular Longevity. (2014) 2014, 360438–31, 10.1155/2014/360438. 24999379 PMC4066722

[9] Prior R. L. , Wu X. , and Schaich K. , Standardized Methods for the Determination of Antioxidant Capacity and Phenolics in Foods and Dietary Supplements, Journal of Agricultural and Food Chemistry. (2005) 53, no. 10, 4290–4302, 10.1021/jf0502698. 15884874

[10] Niki E. , Assessment of Antioxidant Capacity in Vitro and in Vivo, Free Radical Biology and Medicine. (2010) 49, no. 4, 503–515, 10.1016/j.freeradbiomed.2010.04.016. 20416370

[11] Kotebagilu N. P. , Palvai V. R. , and Urooj A. , Ex Vivo Antioxidant Activity of Selected Medicinal Plants Against Fenton Reaction-Mediated Oxidation of Biological Lipid Substrates, Biochemistry Research International. (2015) 2015, 10.1155/2015/728621. PMC4735996 26933511

[12] Xicoy H. , Brouwers J. F. , Kalnytska O. , Wieringa B. , and Martens G. J. M. , Lipid Analysis of the 6-Hydroxydopamine-Treated SH-SY5Y Cell Model for Parkinson’s Disease, Molecular Neurobiology. (2020) 57, no. 2, 848–859, 10.1007/s12035-019-01733-3. 31493240 PMC7031185

[13] Afifi S. M. , Gök R. , Eikenberg I. et al., Comparative Flavonoid Profile of Orange (*Citrus sinensis*) Flavedo and Albedo Extracted by Conventional and Emerging Techniques Using UPLC-IMS-MS, Chemometrics and Antioxidant Effects, Frontiers in Nutrition. (2023) 10, 10.3389/fnut.2023.1158473. PMC10279959 37346911

[14] Saini R. K. , Ranjit A. , Sharma K. et al., Bioactive Compounds of Citrus Fruits: A Review of Composition and Health Benefits of Carotenoids, Flavonoids, Limonoids, and Terpenes, Antioxidants (Basel, Switzerland). (2022) 11, no. 2, 10.3390/antiox11020239. PMC8868476 35204122

[15] Ferrer V. , Paymal N. , Quinton C. , Tomi F. , and Luro F. , Investigations of the Chemical Composition and Aromatic Properties of Peel Essential Oils Throughout the Complete Phase of Fruit Development for Two Cultivars of Sweet Orange (*Citrus sinensis* (L.) Osb.), Plants. (2022) 11, no. 20, 10.3390/plants11202747. PMC9610080 36297771

[16] Machado C. A. T. , Hodel K. V. S. , Lepikson H. A. , and Machado B. A. S. , Distillation of Essential Oils: An Innovative Technological Approach Focused on Productivity, Quality and Sustainability, PLoS One. (2024) 19, no. 2, 10.1371/journal.pone.0299502. PMC10903824 38421961

[17] Ashmawy N. S. , Nilofar N. , Zengin G. , and Eldahshan O. A. , Metabolic Profiling and Enzyme Inhibitory Activity of the Essential Oil of Citrus Aurantium Fruit Peel, BMC Complementary Medicine and Therapies. (2024) 24, no. 1, 10.1186/s12906-024-04505-2. PMC11238441 38987702

[18] Chen G. W. , Lin Y. H. , Lin C. H. , and Jen H. C. , Antibacterial Activity of Emulsified Pomelo (Citrus Grandis Osbeck) Peel Oil and Water-Soluble Chitosan on Staphylococcus aureus and Escherichia coli, Molecules (Basel, Switzerland). (2018) 23, no. 4, 10.3390/molecules23040840. PMC6017636 29642399

[19] Costa-Oliveira C. D. , Ramos Y. J. , Queiroz G. A. , Guimarães E. F. , Viçosa A. L. , and Moreira D. L. , Essential Oils from Piper lhotzkyanum Kunth Leaves From Brazilian Atlantic Forest: Chemical Composition and Stability in Different Storage Conditions, Journal of Oleo Science. (2021) 70, no. 7, 995–1005, 10.5650/jos.ess20332. 34121027

[20] Ito F. , Sono Y. , and Ito T. , Measurement and Clinical Significance of Lipid Peroxidation as a Biomarker of Oxidative Stress: Oxidative Stress in Diabetes, Atherosclerosis, and Chronic Inflammation, Antioxidants (Basel, Switzerland). (2019) 8, no. 3, 10.3390/antiox8030072. PMC6466575 30934586

[21] Rizzardi N. , Pezzolesi L. , Samorì C. et al., Natural Astaxanthin is a Green Antioxidant Able to Counteract Lipid Peroxidation and Ferroptotic Cell Death, International Journal of Molecular Sciences. (2022) 23, no. 23, 10.3390/ijms232315137. PMC9737268 36499464

[22] Pieńkowska N. , Fahnestock M. , Mahadeo C. et al., Induction of Oxidative Stress in SH-SY5Y Cells by Overexpression of hTau40 and its Mitigation by Redox-Active Nanoparticles, International Journal of Molecular Sciences. (2022) 24, no. 1, 10.3390/ijms24010359. PMC9820486 36613801

[23] Mairuae N. , Jittiwat J. , Apaijit K. et al., Synergistic Effects of Anthocyanin-Enriched *Morus Alba* L. Extract and Vitamin C: Promising Nutraceutical Ingredients in Functional Food Development for Neuroprotection, Foods. (2025) 14, no. 21, 10.3390/foods14213630. PMC12610446 41227604

[24] López P. L. , Juncos N. S. , Grosso N. R. , and Olmedo R. H. , *Minthostachys mollis* Essential Oil and its Combination With Tert-Butylhydroquinone for Control of Lipid Oxidation, European Journal of Lipid Science and Technology. (2022) 124, no. 11, 10.1002/ejlt.202200081.

[25] Juncos N. S. , Ponso C. F. C. , Grosso N. R. , and Olmedo R. H. , Oxidation Protection Efficiency of the Combination of *Minthostachys mollis* K. and *Origanum vulgare* L. Essential Oils With “Chain-Breaking” and “Termination-Enhancing” Antioxidant Mechanisms, Journal of Food Science. (2024) 89, no. 12, 9166–9178, 10.1111/1750-3841.17546. 39581625

[26] Juncos N. S. , Corradi M. P. , Cravero Ponso C. F. , and Olmedo R. H. , Evaluation of Genus *Cymbopogon* as Potential Antioxidant in Foods Using Vacuum-Packed and Non-Vacuum-Packed in Roasted Peanuts as a Model to Assess Their Oxidative Stability and Shelf Life, Food and Humanity. (2026) 6, 10.1016/j.foohum.2026.100994.

[27] Ma Q. , Role of Nrf2 in Oxidative Stress and Toxicity, Annual Review of Pharmacology and Toxicology. (2013) 53, no. 1, 401–426, 10.1146/annurev-pharmtox-011112-140320. PMC4680839 23294312

[28] Tonelli C. , Chio I. I. C. , and Tuveson D. A. , Transcriptional Regulation by Nrf2, Antioxidants & Redox Signaling. (2018) 29, no. 17, 1727–1745, 10.1089/ars.2017.7342. 28899199 PMC6208165

